# Clinical and laboratory characteristics and management in patients diagnosed with multiple myeloma: real-world cohort in a Peruvian referral clinic

**DOI:** 10.3332/ecancer.2026.2127

**Published:** 2026-05-20

**Authors:** Victor Rivera Francia, Alejandro Leon Garrido-Lecca, Virgilio E Failoc-Rojas, Robert Villacorta, Yesenia Huerta-Collado, Carlos Carracedo, Marco Villena-Lazo, Luis Casanova Marquez

**Affiliations:** 1 ALIADA Centro Oncológico, Lima 15036, Peru; 2 Universidad San Ignacio de Loyola, Lima 12175, Peru

**Keywords:** autologous cell transplantation, multiple myeloma, Peru, survival

## Abstract

**Background:**

Multiple myeloma (MM) is a malignant proliferation of clonal plasma cells causing bone lesions, renal impairment, anemia and hypercalcemia. Real-world data on the clinical characteristics, treatment patterns and outcomes of patients with MM in Latin America, particularly in Peru, remain scarce. This study aimed to describe the clinical and laboratory features, characteristics, treatment and 5-year survival of MM patients in a Peruvian referral clinic.

**Methods:**

We conducted a retrospective cohort study including adult patients diagnosed with MM (ICD-10 C90.0) between 2016 and 2020, identified from institutional clinical records. Demographic, clinical, laboratory and treatment data were collected. Overall survival was estimated using Kaplan–Meier.

**Results:**

Among transplant recipients, women predominated (52.2%), whereas men predominated in the total cohort (52.4%). The mean age was 55.5 years for transplant patients and 60.6 for all patients. Eastern Cooperative Oncology Group (ECOG) 1 was most common (52.2%) and stage III was frequent (48.9% transplant; 47.3% overall). Vertebral lesions were predominant (69.6% transplant; 65.5% overall). Bortezomib-lenalidomide-dexamethasone (VRd) was the main first-line regimen (69.6% and 68.3%, respectively). Five-year survival was 87.9% for transplant recipients and 64.8% overall.

**Conclusion:**

MM patients commonly presented with advanced stage, vertebral lesions, ECOG 1 and received VRd; transplant recipients achieved excellent survival.

## Introduction

Multiple myeloma (MM) is a malignant neoplasm characterised by the proliferation of clonal plasma cells within the bone marrow. This abnormal expansion leads to the excessive production of monoclonal (M) immunoglobulins, resulting in a range of clinical manifestations, including osteolytic bone lesions, anemia, hypercalcemia and impair renal function [1]. The etiology of MM is multifactorial, with advance age, genetic predisposition and environmental factors, such as obesity, being the most significant risk factors [2].

Although MM accounts for only 1% of malignancies; it is the second most common hematologic cancer after lymphoma [1, 2]. Globally, over 140,000 new cases are diagnosed annually, highlighting its growing incidence [3]. In Peru, the most recent epidemiological registry, reported by the National Institute of Neoplastic Diseases estimated an incidence of 1.2 cases per 100,000 invidividuals [4].

Diagnosis of MM relies on the detection of M proteins in biological fluids, assessed through serum protein electrophoresis, serum immunofixation and free light chain assay. The presence of M protein is defined as serum levels ≥1 g/dL or urine levels ≥200 mg/dL in urine [5, 6].

Advances in treatment have significantly improved survival rates, with therapeutic strategies tailored to patient-specific factors such as age, comorbidities and genetic markers. Standard therapies include chemotherapy, proteasome inhibitors, immunomodulatory agents and autologous stem cell transplantation. Additionally, novel immunotherapies, such as M antibodies targeting PD-1 and PD-L1, have been developed to enhance immune-mediated tumour suppression [7].

Prognostic factors in MM include age, disease stage at diagnosis, cytogenetic abnormalities and response to initial therapy. Notably, patients older than 50 years exhibit significantly reduced median survival compared to younger individuals [8, 9].

The Peruvian population may have an impact on MM biology and treatment response. Furthermore, treatment availability and access to healthcare may vary between Peru and other countries, potentially influencing patient outcomes. Furthermore, identifying risk factors specific to the Peruvian population could help to develop better MM prevention and management strategies. Investigating MM in this specific demographic is crucial to optimising management strategies, improving patient outcomes and enhancing the overall quality of life for affected individuals. In addition, we explored differences in clinical characteristics, treatment patterns and survival outcomes between patients who underwent autologous stem cell transplantation and those who did not.

## Materials and methods

### Study design

This retrospective, real-world cohort study assessed the 5-year overall survival (OS) of patients diagnosed with MM who attended Aliada Clinic, a referral oncology center in Lima, Peru, between 2016 and 2020. Comparative analyses were performed between patients who underwent autologous stem cell transplantation and those who did not.

### Population and sample

The study population included adult patients (≥18 years) diagnosed with MM based on the International Myeloma Working Group criteria [10]. Eligible patients had received first-line regimens with bortezomib-lenalidomide-dexamethasone (VRd) or bortezomib-thalidomide-dexamethasone (VTD).

Patients with a diagnosis of another hematologic malignancy, M gammopathy of uncertain significance, solitary plasmacytoma or other MM differential were excluded from the study. Additionally, pregnant or lactating women were excluded from participation. Patients with insufficient data for the study variables of interest were also excluded.

### Procedures

Patients diagnosed with MM were identified through medical records coded under ICD-10 C90.0. Data were collected retrospectively over a 5-year follow-up period.

### Instrument and variables

The data for the study were gathered using a form, which inquired about epidemiologic (age (<60 years or ≥ 60 years) and sex) and clinical-oncologic parameters, variables (stage at diagnosis (0–2) and bone lesion area (spine, ribs, skull and so on)). The data collection form also inquired about long bone epiphysis, hemoglobin (anemic and non-anemic), albumin (low, normal and high), globulin (low, normal and high), lactate dehydrogenase ((LDH); altered and non-altered) and B2 microglobulin as well as the predominant immunoglobulin A (IgA), G (IgG) and M (IgM), the International Staging System (ISS) (I–III), creatinine on admission and the negative predictive value (NPV) assessed at diagnosis or during treatment. Functional status was assessed using the Eastern Cooperative Oncology Group (ECOG) performance status scale (0 to 4 on admission).

Therapeutic data included first-line regimen (VRd or VTD). The variables included the number of first-line cycles, whether progression to first-line therapy was observed (yes/no), the second-line therapy used (daratumumab or other), the patient’s status at the time of data collection (alive or dead) and the OS in years.

### Statistical analysis

Data were initially recorded in Microsoft Excel^®^ spreadsheet and subsequently analysed using the statistical program STATA v19.0 (StataCorp, TX, USA).

Descriptive statistics were presented as frequencies and percentages (categorical variables) or measures of central tendency and dispersion (continuous variables). Associations between categorical variables and outcomes (OS or disease progression) were evaluated using the chi-squared test or Fisher’s exact test as appropriate. Continuous variables were compared using *t*-tests or Mann–Whitney *U* tests based on normality assessments.

OS was estimated using the Kaplan–Meier method, and survival curves were compared using the log-rank test. Cox proportional hazards regression models were used to estimate crude and adjusted hazard ratios (HRs) with 95% confidence intervals (CIs) for mortality and disease progression.

Prior to multivariable analysis, we assessed collinearity between independent variables using the variance inflation factor (VIF). Variables with high collinearity (VIF >10) were excluded from the adjusted models to ensure model stability and interpretability. The final multivariable model was selected based on clinical relevance and statistical significance in univariable analyses.

The proportional hazards assumption was evaluated using Schoenfeld residuals and log–log survival plots. Model adequacy and fit were verified through visual inspection of Cox-Snell residuals. Statistical significance was set at a *p*-value <0.05.

Figures in Kaplan-Meier survival curves for OS and stratified by transplantation status were generated to support interpretation of the results.

### Ethical aspects

The study underwent a rigorous ethical review by the Ethics Committee of Clinica Aliada, Peru and subsequently approved with ethical number ACO-2022-04CEI. Throughout the study, the ethical principles outlined in the Declaration of Helsinki were adhered to. Informed consent was obtained from adult participants. The principle of anonymity was upheld throughout the study, with participants registered using randomly generated codes. Moreover, only data collected for research purposes were used.

## Results

A total of 84 medical records were reviewed, of which 77 patients met the inclusion criteria. Among them, 42 (54.5%) underwent autologous bone marrow transplantation.

The sex-based distribution revealed that the transplanted patients showed a slight female predominance, with 52.4% of women and 47.6% of men. This trend was reversed among the non-transplanted patients, where 60.0% were men and 40.0% were women. The mean age of the transplanted group was 55.6 years (SD = 7.4), significantly lower than the non-transplanted group, which had a mean age of 67.0 years (SD = 10.1). Overall, 48.1% of patients were younger than 60 years, with the majority of these (66.7%) undergoing transplantation.

Regarding functional status at diagnosis, most patients had an ECOG performance status of 1 (50.0% in the transplanted group and 51.4% in the non-transplanted group), followed by ECOG 2 and 0. Clinical stage at diagnosis showed a predominance of ISS stage I (64.3% of transplanted and 62.9% of non-transplanted patients), followed by stage II. Bone lesions were most frequently located in the vertebral column (69.0% among transplanted patients and 57.1% among non-transplanted).

Baseline laboratory parameters were generally comparable between groups. Baseline hemoglobin <12 g/dL was present in 45.2% of transplanted and 45.7% of non-transplanted patients. Low serum albumin (<3.5 g/dL) was more frequent in non-transplanted patients (25.7% versus 9.5%). Conversely, normal serum albumin (≥3.5–5.5 g/dL) was more common in the transplant group (90.5% versus 74.3%). A globulin level <2.5 g/dL was observed in 17.1% of non-transplanted patients and 14.3% of transplanted patients. LDH levels ≥240 U/L were found in 33.3% of the transplant group and 28.6% of non-transplanted patients.

In terms of treatment, the most common first-line regimen was VRd, used in 78.6% of transplant recipients and 72.7% of non-transplant patients. VTD was used in 21.4% and 27.3%, respectively. Following first-line treatment, 80.0% of transplant patients and 73.1% of non-transplanted patients experienced disease progression. At the 5-year mark, survival was notably higher among the transplant group (92.9%) compared to non-transplanted patients (62.9%). The median OS was 56.4 months in the transplant group and 47.4 months in the non-transplant group (Table 1).

When stratifying by clinical and laboratory characteristics, no significant differences in progression rates were observed by sex, ECOG or bone lesion location (Table 2). However, age showed a significant association with both progression and mortality. Patients aged ≥60 years had a significantly higher mean age among those who died (66.98 versus 58.99 years, *p* = 0.005). Additionally, patients with ECOG 2 at diagnosis had a higher mortality rate (52.9%) compared to those with ECOG 0 (5.9%, *p* = 0.042).

Creatinine levels at diagnosis were significantly higher in non-survivors (1.41 versus 0.88 mg/dL, *p* = 0.006), although not in multivariate analysis. No significant differences in survival were observed by ISS stage or baseline hemoglobin, albumin, LDH or immunoglobulin subtype. However, transplantation status showed a significant association with survival. Mortality at 5 years was 7.1% in the transplant group versus 37.1% in the non-transplant group (*p* = 0.018), Table 2.

**Figure 1.**
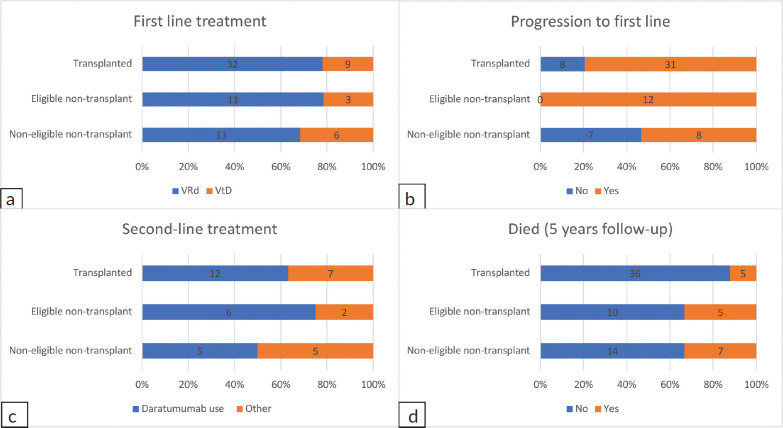
Distribution based on the condition of the population with MM in an oncology clinic in Peru. Based on their condition: (a): distribution of patients who received first-line drugs; (b): patients who progressed to first-line treatment condition; (c): second-line treatment (use of daratumumab) and (d): patients who died (within 5 years).

The distribution of first-line therapies based on clinical conditions of the transplant candidate, transplanted and ineligible patients followed a similar pattern, with VRd having a higher prevalence, followed by VTD. The majority of the transplanted patients received daratumumab as second-line treatment, which was also observed in patients who were ineligible for transplantation (Figure 1).

In the crude analysis, patients aged 60 years or older showed a higher risk of mortality compared to those under 60 (HR = 2.57; 95% CI: 0.92–7.17; *p* = 0.072), although this association did not reach statistical significance and was not included in the adjusted model. Male sex was associated with an increased risk of death in the adjusted analysis (HRa = 3.07; 95% CI: 1.20–7.84; *p* = 0.019), despite the crude HR not being significant (HR = 1.83; *p* = 0.225), suggesting a potential confounding effect of other variables.

Regarding functional status at diagnosis, patients with ECOG scores of 2 had a markedly increased mortality risk compared to those with ECOG 0, both in the crude (HR = 6.96; 95% CI: 0.87–55.83; *p* = 0.068) and adjusted models (HRa = 7.21; 95% CI: 0.82–32.01; *p* = 0.074), although results remained borderline significant.

Laboratory values showed a protective trend for higher serum albumin and globulin levels. Patients with albumin ≥3.5 g/dL had a lower crude risk of death (HR = 0.41; 95% CI: 0.15–1.17; *p* = 0.097), though this was not confirmed in the adjusted model. Similarly, globulin levels between 2.5 and 5.5 g/dL were associated with a lower mortality risk compared to <2.5 g/dL in both the crude (HR = 0.37; 95% CI: 0.14–1.05; *p* = 0.061) and adjusted models (HRa = 0.38; 95% CI: 0.10–1.44; *p* = 0.156). A globulin level ≥5.5 g/dL also showed a strong protective trend in the adjusted analysis (HRa = 0.18; 95% CI: 0.03–1.03; *p* = 0.054), approaching statistical significance. No significant associations were observed for mortality with ISS stage, LDH, hemoglobin or β2-microglobulin levels. Similarly, no difference was noted between first-line regimens (VTD versus VRd) in either model.

Importantly, undergoing autologous bone marrow transplantation was independently associated with a significantly reduced risk of mortality (HRa = 0.30; 95% CI: 0.11–0.82; *p* = 0.019), confirming its role as a favorable prognostic factor at 5 years.

**Figure 2.**
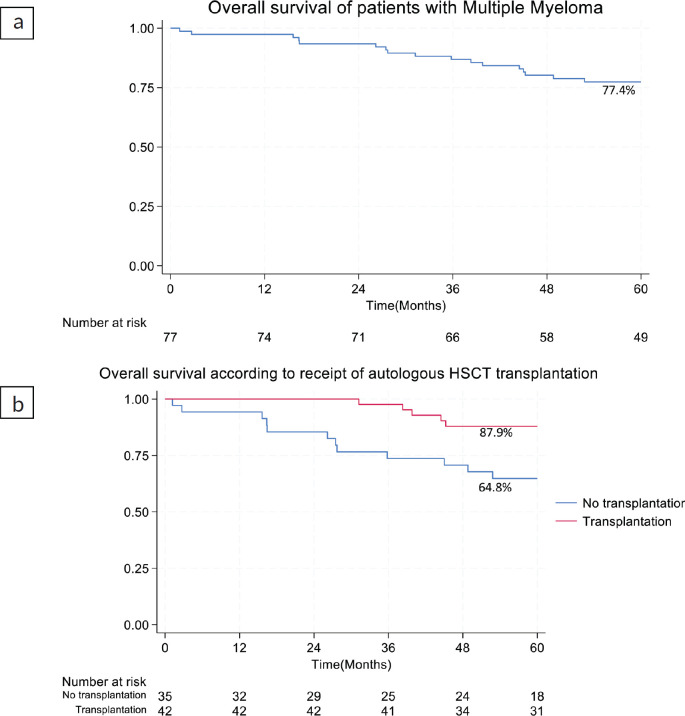
Estimated OS of the MM population in a Peruvian oncology clinic. (a): OS of patients with MM. (b): OS according to receipt of autologous HSCT transplantation.

Kaplan–Meier analysis revealed stable survival curves during the first 24 months, with a pronounced decline after 36 months. Transplanted patients showed significantly better survival than non-transplanted patients (Figure 2).

The survival curve remained stable for the first 24 months; however, a significant decline was observed after 36 months. Survival rates were significantly different between patients who received transplants and those who did not (Figure 2). Five-year survival was 87.9% for transplant recipients and 64.8% overall.

## Discussion

In this study, we found that at the 5-year follow-up, 87.9% transplant patients were alive, whereas 22.6% of the overall population had died. This finding emphasises the potential efficacy of transplantation in improving long-term survival outcomes, which is consistent with previous research demonstrating the superiority of autologous hematopoietic stem cell transplantation in terms of progression-free survival and OS when compared with non-transplantation treatments [11].

In the study, the majority of the patients were of advanced age (≥60 years) and had stage III myeloma, indicating advanced disease. The presence of a spinal lesion is consistent with the previous research that identified the spine as one of the most common sites of bone lesions in patients with MM, contributing to the significant morbidity observed in these patients [12].

The most common immunoglobulin in our sample was IgG, which is consistent with data showing that approximatel 75% of patients with MM have IgG myeloma [13]. Furthermore, the fact that ISS I was the most common in our study population suggests that, despite advanced age and stage-related complications, a large proportion of patients had a lower risk profile at diagnosis. This could have a positive impact on their treatment outcomes.

The VRd regimen is an effective first-line treatment for MM. In a study conducted by Durie *et al* [14] VRd was found to significantly improve progression-free survival and OS when compared with regimens lacking proteasome inhibitors or immunomodulators. The SWOG S0777 study, which compared VRd with lenalidomide and dexamethasone in previously untreated patients, found that VRd had a median progression-free survival of 43 versus 30 months for lenalidomide and dexamethasone. In addition, the OS was improved (73% versus 64% at 4 years) [15].

Furthermore, the VTD regimen has proven effective as an induction therapy. Moreau *et al* [16] found that VTD is associated with a high complete response rate before transplantation, with overall response rates approaching 90%. Although this regimen is less common than VRd in some countries due to the neurologic toxicity associated with thalidomide, it is still a variable option, particularly when lenalidomide is unavailable or less preferred due to safety or cost concerns.

Although both regimens are effective, the choice between VRd and VTD may be influenced by the patient’s tolerance for the specific drugs, the profiles of associated side effects and the availability of the agents. VRd is frequently the preferred option due to its superior comparative efficacy and safety profile, particularly the lower incidence of peripheral neuropathy when compared with regimens containing thalidomide. However, in cases where lenalidomide is prohibitively expensive or unavailable, VTD remains a viable option.

Our findings revealed a relationship between baseline albumin and globulin levels and disease progression, indicating that these biomarkers may serve as useful indicators for assessing disease progression and categorising patients into risk groups. Although there were no statistically significant differences in 5-year mortality, this is most likely due to the study’s small sample size. This parameter is associated with a poorer prognosis and lower survival rates according to findings from a similar Colombian study [17]. Albumin and globulin are markers that are available in Peruvian laboratories and hospitals. Therefore, further investigation of these markers as predictive markers in larger cohorts is warranted.

Our findings revealed a statistically marginal relationship between altered baseline LDH levels and disease progression or 5-year survival. However, Lu *et al* [18] and Fan [19] demonstrated that altered LDH was a risk factor associated with lower median survival in MM patients. It should be noted that these studies involved different populations, such as the one conducted by Lu *et al* [18], patients who had undergone hematopoietic stem cell transplantation or who had a concomitant systemic cardiac, pulmonary or renal decompensated disorder were excluded from the study [19]. Our study, however, did not take these exclusion criteria into account.

The findings of this study should be interpreted considering several limitations. First, the retrospective design may be subject to selection bias and limits the ability to establish casual associations. Second, the relatively small sample size and single-center setting may restrict the generalisability of the results to other populations. Third, heterogeneity in treatment regimens prior to and during the study period, as well as incomplete availability of cytogenetic and molecular risk data, could have influenced survival outcomes. Additionally, the low number of mortality events may have reduced the statistical power to detect significant differences between groups. Despite these limitations, our study provides valuable real-world data on the clinical characteristics, management and outcomes of patients with MM in a Peruvian referral center.

This study has important implications for public health in Peru and other low- and middle-income countries. In these settings, the use of novel agents such as daratumumab is often limited due to high costs and restricted access through public or private insurance systems. Consequently, most patients begin treatment with VRd or VTD regimens, which, although effective, may not offer the same survival benefits as those incorporating M antibodies. By demonstrating the substantial mortality reduction associated with autologous transplantation, this study underscores the need to prioritise timely referral and access to transplant services as a cost-effective strategy to improve outcomes in resource-constrained health systems.

## Conclusion

In conclusion, the most common characteristics of the patients evaluated were female sex, advanced stage of diagnosis (III), location of bone lesion in the spine, ECOG performance status 1, ISS 1, first-line treatment VRd and second-line treatment daratumumab. Within 5 years, 20.8% of the overall population had passed away. Baseline globulin levels, and ECOG 0, may be used as prognostic markers, and transplantation has been shown to reduce 5-year mortality.

## Conflicts of interest

None of the authors had competing personal, financial, commercial or academic interests.

## Funding

There was no financial support for this research.

## Informed consent

This study was carried out in accordance with the ethical standards of the responsible institution for human subjects, as well as with the Declaration of Helsinki.

## Consent for publication

The manuscript has not been submitted for publication or consideration elsewhere.

## Availability of data and materials

All data generated or analysed during this study are included in this article.

## Author contributions

Conceptualisation: VRF, ALGL, MVL and LCM. Methodology: VEFR, RV, YHC, CC. Initial manuscript draft: VEFR, VRF,LCM. Data Analysis: VEFR, CC. Interpretation: All authors. Writing - Review and Editing: All authors. All authors read and approved the final version of the manuscript.

## Figures and Tables

**Table 1.** General and laboratory characteristics and treatment in patients with MM visiting a Peruvian oncology clinic.

Characteristic	Non-transplant patients (n = 35; 45.5%)	Transplant patients (n = 42; 54.5%)	Total patients (n = 77; 100%)
Sex			
Female	14 (40.0%)	22 (52.4%)	36 (46.8%)
Male	21 (60.0%)	20 (47.6%)	41 (53.2%)
Age (years)	66.97 (10.1)	55.58 (7.4)	60.76 (10.4)
Age (categorized)			
<60 years	9 (25.7%)	28 (66.7%)	37 (48.1%)
≥60 years	26 (74.3%)	14 (33.3%)	40 (51.9%)
ECOG (debut)			
0	6 (17.1%)	9 (21.4%)	15 (19.5%)
1	18 (51.4%)	21 (50.0%)	39 (50.6%)
2	11 (31.4%)	12 (28.6%)	23 (29.9%)
Bone lesion zone			
Vertebral column	20 (57.1%)	29 (69.0%)	49 (63.6%)
Ribs	2 (5.7%)	4 (9.5%)	6 (7.8%)
Cranium	3 (8.6%)	3 (7.1%)	6 (7.8%)
Epiphyses of long bones	2 (5.7%)	4 (9.5%)	6 (7.8%)
Pelvis	7 (20.0%)	2 (4.8%)	9 (11.7%)
Basal hemoglobin (g/dL)			
<12	16 (45.7%)	19 (45.2%)	35 (45.5%)
≥12–15	19 (54.3%)	23 (54.8%)	42 (54.5%)
Basal albumin (g/dL)			
<3.5	9 (25.7%)	4 (9.5%)	13 (16.9%)
≥3.5–5.5	26 (74.3%)	38 (90.5%)	64 (83.1%)
Basal globulin (g/dL)			
<2.5	6 (17.1%)	6 (14.3%)	12 (15.6%)
2.5–5.5	21 (60.0%)	31 (73.8%)	52 (67.5%)
≥5.5	8 (22.9%)	5 (11.9%)	13 (16.9%)
LDH basal (U/L)			
<240	25 (71.4%)	28 (66.7%)	53 (68.8%)
≥240–400	10 (28.6%)	14 (33.3%)	24 (31.2%)
B2 basal microglobulin (mg/L)			
0.8–3.5	22 (62.9%)	28 (66.7%)	50 (64.9%)
3.5–9	13 (37.1%)	14 (33.3%)	27 (35.1%)
Predominant immunoglobulin			
IgA	3 (10.7%)	8 (25.0%)	11 (18.3%)
IgG	25 (89.3%)	20 (62.5%)	45 (75.0%)
IgM	0 (0.0%)	4 (12.5%)	4 (6.7%)
ISS			
1	22 (62.9%	27 (64.3%)	49 (63.6%)
2	10 (28.6%)	14 (33.3%)	24 (31.2%)
3	3 (8.6%)	1 (2.4%)	4 (5.2%)
Creatine at admission	0.87 (0.2)	1.05 (0.6)	0.95 (0.5)
NPV	17.45 (33.7)	59.13 (184.1)	38.29 (132.7)
First-line treatment			
VRd	24 (72.7%)	33 (78.6%)	57 (76.0%)
VTD	9 (27.3%)	9 (21.4%)	18 (24.0%)
Response to first treatment			
Response	7 (26.9%)	8 (20.0%)	15 (22.7%)
Progression	19 (73.1%)	32 (80.0%)	51 (77.3%)
Deceased (5 years)			
Alive	22 (62.9%)	39 (92.9%)	61 (79.2%)
Died	13 (37.1%)	3 (7.1%)	16 (20.8%)
Median survival (months)	47.4 (19.2)	56.4 (7.4)	52.4 (14.5)

ECOG, Eastern Cooperative Oncology Group; ISS, International Staging System; LDH, lactate dehydrogenase; NPV, negative predictive value

**Table 2.** Disease progression and OS at 5 years in patients with MM visiting a Peruvian oncology clinic.

	Progression at first treatment			OS at 5 years		
Characteristic	**No**	**Yes**	***p* value**	**Survived**	**Died**	***p* value**
Sex						
Female	6 (40.0%)	25 (49.0%)	0.538	30 (50.0%)	6 (35.3%)	0.283
Male	9 (60.0%)	26 (51.0%)		30 (50.0%)	11 (64.7%)	
Age (categorized)						
<60 years	5 (33.3%)	28 (54.9%)	0.142	32 (53.3%)	5 (29.4%)	0.081
≥60 years	10 (66.7%)	23 (45.1%)		28 (46.7%)	12 (70.6%)	
Age (years)	65.82 (12.1)	58.89 (9.6)	0.016	58.99 (9.6)	66.98 (11.0)	0.005
ECOG (debut)						
0	2 (13.3%)	11 (21.6%)	0.753	14 (23.3%)	1 (5.9%)	0.042
1	8 (53.3%)	23 (45.1%)		32 (53.3%)	7 (41.2%)	
2	5 (33.3%)	17 (33.3%)		14 (23.3%)	9 (52.9%)	
Bone lesion zone						
Vertebral column	9 (60.0%)	33 (64.7%)	0.816	37 (61.7%)	12 (70.6%)	0.667
Ribs	2 (13.3%)	4 (7.8%)		6 (10.0%)	0 (0.0%)	
Cranium	1 (6.7%)	4 (7.8%)		5 (8.3%)	1 (5.9%)	
Epiphyses of long bones	2 (13.3%)	3 (5.9%)		4 (6.7%)	2 (11.8%)	
Pelvis	1 (6.7%)	6 (11.8%)		7 (11.7%)	2 (11.8%)	
Basal hemoglobin (g/dL)						
<12	6 (40.0%)	27 (52.9%)	0.378	27 (45.0%)	8 (47.1%)	0.880
≥12–15	9 (60.0%)	24 (47.1%)		33 (55.0%)	9 (52.9%)	
Basal albumin (g/dL)						
<3.5	3 (20.0%)	6 (11.8%)	0.414	8 (13.3%)	5 (29.4%)	0.118
≥3.5–5.5	12 (80.0%)	45 (88.2%)		52 (86.7%)	12 (70.6%)	
Basal globulin (g/dL)						
<2.5	3 (20.0%)	9 (17.6%)	0.581	7 (11.7%)	5 (29.4%)	0.196
2.5–5.5	11 (73.3%)	33 (64.7%)		42 (70.0%)	10 (58.8%)	
≥5.5	1 (6.7%)	9 (17.6%)		11 (18.3%)	2 (11.8%)	
LDH basal (U/L)						
<240	8 (53.3%)	39 (76.5%)	0.082	43 (71.7%)	10 (58.8%)	0.313
≥240–400	7 (46.7%)	12 (23.5%)		17 (28.3%)	7 (41.2%)	
B2 basal microglobulin (mg/L)						
0.8–3.5	7 (46.7%)	33 (64.7%)	0.209	42 (70.0%)	8 (47.1%)	0.080
3.5–9	8 (53.3%)	18 (35.3%)		18 (30.0%)	9 (52.9%)	
Predominant immunoglobulin						
IgA	2 (25.0%)	7 (16.3%)	0.653	8 (16.7%)	3 (25.0%)	0.754
IgG	6 (75.0%)	33 (76.7%)		37 (77.1%)	8 (66.7%)	
IgM	0 (0.0%)	3 (7.0%)		3 (6.2%)	1 (8.3%)	
ISS						
1	7 (46.7%)	33 (64.7%)	0.160	41 (68.3%)	8 (47.1%)	0.255
2	8 (53.3%)	15 (29.4%)		16 (26.7%)	8 (47.1%)	
3	0 (0.0%)	3 (5.9%)		3 (5.0%)	1 (5.8%)	
Creatine at admission	1.41 (0.9)	0.88 (0.4)	0.006	0.99 (0.5)	0.83 (0.2)	0.319
NPV	19.40 (34.4)	50.69 (160.8)	0.590	41.19 (147.7)	26.97 (38.8)	0.755
First-line treatment						
VRd	9 (60.0%)	42 (82.4%)	0.069	45 (77.6%)	12 (70.6%)	0.552
VTD	6 (40.0%)	9 (17.6%)		13 (22.4%)	5 (29.4%)	
Transplante						
Non-transplant	7 (46.7%)	19 (37.3%)	0.512	23 (38.3%)	12 (70.6%)	0.018
Transplant patients	8 (53.3%)	32 (62.7%)		37 (61.7%)	5 (29.4%)	

ECOG, Eastern Cooperative Oncology Group; ISS, International Staging System; LDH, lactate dehydrogenase; N.A., not applicable

**Table 3.** Association of characteristics and OS at 5 years in patients with MM visiting a Peruvian oncology clinic.

	Mortality at 5 years			Mortality at 5 years		
	**HRc**	**95% CI**	***p* value**	**HRa**	**95% CI**	***p* value**
Age (years)						
<60	Ref.					
≥60	2.57	0.92–7.17	0.072	N.A.		
Sex						
Female	Ref.			Ref.		
Male	1.83	0.69–4.89	0.225	3.07	1.20–7.84	0.019
ECOG (debut)						
0	Ref.			Ref.		
1	3.01	0.36–25.01	0.308	3.80	0.49–29.71	0.204
2	6.96	0.87–55.83	0.068	7.21	0.82–32.01	0.074
Basal hemoglobin (g/dL)						
<12	Ref.			Ref.		
≥12–15	0.93	0.36–2.40	0.885	2.83	0.57–14.02	0.204
Basal albumin (g/dL)						
<3.5	Ref.			Ref.		
≥3.5–5.5	0.41	0.15–1.17	0.097	0.84	0.08–8.71	0.884
Basal globuline (g/dL)						
< 2.5	Ref.			Ref.		
2.5–5.5	0.37	0.14–1.05	0.061	0.38	0.10–1.44	0.156
≥5.5	0.32	0.56–1.78	0.193	0.18	0.03–1.03	0.054
Albumin/Globulin ratio						
Low (<1)	Ref.					
Normal (≥1)	1.01	0.40–2.61	0.985	N.A.		
LDH basal (U/L)						
<240	Ref.			Ref.		
≥240–400	1.56	0.60–4.06	0.361	1.38	0.52–3.65	0.521
B2 basal microglobulina (mg/L)						
0.8–3.5	Ref.			Ref.		
3.5–9	2.36	0.92–6.07	0.076	2.65	0.67–10.39	0.163
ISS						
1	Ref.					
2	2.35	0.89–6.27	0.087	N.A.		
3	1.46	0.21–9.91	0.701			
First-line treatment						
VRd	Ref.			Ref.		
VTD	1.29	0.47–3.54	0.624	1.15	0.29–4.62	0.844
Transplante						
Non-transplant	Ref.			Ref.		
Transplant patients	0.29	0.10–0.79	0.016	0.30	0.11– 0.82	0.019

ECOG, Eastern Cooperative Oncology Group; HRa, hazard ratio adjusted; HRc, hazard ratio crude; ISS, International Staging System; LDH, lactate dehydrogenase; N.A., not applicable. Ref: Grupe reference

## References

[1] Kazandjian D (2016). Multiple myeloma epidemiology and survival: a unique malignancy. Semin Oncol.

[2] Lazaris V, Hatziri A, Symeonidis A (2021). The lipoprotein transport system in the pathogenesis of multiple myeloma: advances and challenges. Front Oncol.

[3] Hemminki K, Försti A, Houlston R (2021). Epidemiology, genetics and treatment of multiple myeloma and precursor diseases. Int J Cancer.

[4] Departamento de Oncología Médica, Instituto Nacional de Enfermedades Neoplasicas (2013). Guía de práctica clínica de mieloma múltiple.

[5] Rajkumar SV (2020). Multiple myeloma: 2020 update on diagnosis, risk-stratification and management. Am J Hematol.

[6] Segura MK, Oquendo ER, Barrera CJ (2020). Mieloma Múltiple: Métodos de Diagnóstico. Am Health.

[7] Goldschmidt H, Ashcroft J, Szabo Z (2019). Navigating the treatment landscape in multiple myeloma: which combinations to use and when?. Ann Hematol.

[8] Bębnowska D, Hrynkiewicz R, Grywalska E (2021). Immunological prognostic factors in multiple myeloma. Int J Mol Sci.

[9] Wallington-Beddoe CT, Mynott RL (2021). Prognostic and predictive biomarker developments in multiple myeloma. J Hematol Oncol.

[10] Rajkumar SV, Dimopoulos MA, Palumbo A (2014). International myeloma working group updated criteria for the diagnosis of multiple myeloma. Lancet Oncol.

[11] Attal M, Lauwers-Cances V, Hulin C (2015). Autologous transplantation for multiple myeloma in the era of new drugs: a phase III study of the Intergroupe Francophone du Myelome (IFM/DFCI 2009 Trial). Blood.

[12] Terpos E, Morgan G, Dimopoulos MA (2013). International myeloma working group recommendations for the treatment of multiple myeloma-related bone disease. J Clin Oncol.

[13] Dimopoulos MA, Moreau P, Terpos E (2022). Multiple myeloma: EHA-ESMO clinical practice guidelines for diagnosis, treatment and follow-up. Ann Oncol.

[14] Durie BGM, Hoering A, Abidi MH (2017). Bortezomib with lenalidomide and dexamethasone versus lenalidomide and dexamethasone alone in patients with newly diagnosed myeloma without intent for immediate autologous stem-cell transplant (SWOG S0777): a randomised, open-label, phase 3 trial. Lancet.

[15] Stewart AK, Rajkumar SV, Dimopoulos MA (2015). Carfilzomib, Lenalidomida, y dexamethasone for relapsed multiple myeloma. N Engl J Med.

[16] Moreau P, Hulin C, Macro M (2016). VTD is superior to VCD prior to intensive therapy in multiple myeloma: results of the prospective IFM2013–04 trial. Blood.

[17] Atencia-Flórez C, Quintero-Valencia C, Mondragón-Arismendy M (2023). Clinical, laboratory, cytometry and cytogenetic characteristics of a cohort of patients diagnosed with multiple myeloma for the first time in a third-level hospital in Medellín, Colombia, survival after 8 years of follow-up. Int J Hematol Oncol Stem Cell Res.

[18] Lu W, Xu S, Tan S (2023). Comprehensive analysis and establishment of a prognostic model based on non-genetic predictors in multiple myeloma. Cancer Biomark.

[19] Fan H (2023). Current treatment paradigm and survival outcomes among patients with newly diagnosed multiple myeloma in China: a retrospective multicenter study. Cancer Biol Med.

